# Who gets treated for an eating disorder? Implications for inference based on clinical populations

**DOI:** 10.1186/s12889-024-19283-2

**Published:** 2024-07-02

**Authors:** Alison E. Field, Hannah N. Ziobrowski, Kamryn T. Eddy, Kendrin R. Sonneville, Tracy K. Richmond

**Affiliations:** 1 Department of Epidemiology, Brown University School of Public Health, Box G-S121-2, Providence, RI 02912 USA; 2 Department of Psychiatry, Massachusetts General Hospital, Boston, MA USA; 3 Department of Nutrition, University of Michigan, Ann Arbor, MA USA; 4 Division of Adolescent and Young Adult Medicine, Boston Children’s Hospital, Boston, MA USA

**Keywords:** Eating disorder, Treatment, Weight status, Comorbidity

## Abstract

**Background:**

The minority of people with an eating disorder receive treatment. Little is known about predictors of receiving treatment.

**Methods:**

Using data from the Growing Up Today Study we identified correlates of receiving treatment for an eating disorder among the 1237 U.S. women who answered questions on treatment history in 2013 and reported meeting criteria for subthreshold eating disorder in ≥ 1 year between 1996 and 2013. Logistic regression models using generalized estimating equations were used to estimate the relative odds of receiving treatment.

**Results:**

Approximately 11% of the women reported receiving treatment for an eating disorder. Independent of type of eating disorder, those who had received a diagnosis of depression or anxiety were more likely (odds ratio (OR) = 3.05 95% confidence interval (CI) 1.87–4.97) to receive treatment for an eating disorder. Women with obesity were approximately 85% less likely to receive treatment (OR = 0.13, 95% CI 0.04–0.46) regardless of their type of eating disorder or history of depression of anxiety diagnosis.

**Conclusions:**

Most women meeting criteria for an eating disorder do not receive treatment. Women with BED or obesity are the least likely to receive treatment.

Even in high income countries, [1] most people with a mental health disorder do not receive treatment. [1–3] Reasons for not seeking treatment include cost and other structural barriers, as well as attitudinal barriers. [4] Therefore, the subgroup of people who receive treatment may be a biased sample of the population with a mental health disorder. As a result, inference and generalizability of findings from research conducted using treatment seeking populations is unclear. This issue is particularly true of eating disorders research, because eating disorders are far less likely to be treated relative to other common mental health conditions such as depression and anxiety. [3].

Several studies have found that African American and Hispanic individuals are less likely to seek treatment for an eating disorder. [5, 6] There are numerous reasons for the disparity, including stigma around mental health, a lack of perception of need for treatment on the part of the individual or a health care provider, and treatment costs. Despite the high prevalence, there is widespread stigma around mental health disorders. A recent meta-analysis reported that mental health stigma was higher in racial minorities. [7] This finding could be at least partially confounded by socio-economic status since poverty is more common among some ethnic and racial minority groups and women from more affluent backgrounds are more likely than their peers to perceive a need for treatment [8] and to receive specialized treatment for a mental illness. [1].

Another barrier to receiving treatment is having a clinician identify the disorder. Eating disorders in Mexican American and Black women are under-detected by clinicians, [6, 9] possibly reflecting that many clinicians may erroneously believe that only White women are at high risk. This belief may then be reinforced by the observation that most of the cases seeking specialized treatment are White women from high socio-economic backgrounds, [8] which may reflect that this is the group most likely to be diagnosed and most able to afford treatment.

Another misperception that could result in under diagnosis for eating disorders is weight status. Anorexia nervosa, the least prevalent eating disorder, is defined by a significant low weight. [10] However, individuals with higher weights are the most likely to have an eating disorder. [11, 12] In fact, people with bulimia nervosa and binge-eating disorder are more likely than their peers without eating disorders to have higher weights. [12] While weight loss in an individual with low weights may might prompt a clinician to evaluate the cause of weight change, thus detecting an eating disorder, this same level of attentiveness may not be offered to an individual with obesity. Rather than queried to detect an eating disorder, higher weight patients might instead be evaluated or referred for weight loss treatment. Indeed, research has found that people with eating disorders are more likely to have received treatment for weight loss than an eating disorder. [13, 14].

Many eating disorder research studies focus on treatment-seeking patients and/or are restricted to identifying cases from clinical registers or medical records/databases. Whether the results from these studies can be generalized to the majority of people with an eating disorder who do not receive treatment has not been evaluated. The goal of the current study was to ascertain how eating disorder symptom profiles, weight status, race/ethnic group, socio-economic status (SES), and comorbid psychopathology are related to whether adolescent or young adult women with an eating disorder receives treatment. We hypothesized that women who have a low weight, are White, and middle to high SES would be more likely to be identified as cases, as would women who have received a diagnosis for another form of mental illness and therefore have been evaluated for psychopathology.

## Methods

### Sample

The Growing Up Today Study (GUTS) was established in 1996 by recruiting 9 to 15 year old children who were offspring of women participating in the Nurses' Health Study II, an ongoing cohort study of nurses in the United States. We wrote a detailed letter to the mothers, explaining that the purpose of GUTS was to study the diet, activity, and weight change during adolescence and sought parental consent to enroll their children. We mailed letters and baseline questionnaires to the 13,261 girls and 13,504 boys whose mothers had provided informed consent to invite them to participate in the Growing Up Today Study. The invitation letter and baseline questionnaire were mailed to the child. Approximately 68% of the girls (*n* = 9,039) and 58% of the boys (*n* = 7,843) returned completed questionnaires, thereby assenting to participate in the cohort. Study participants were sent questionnaires annually in 1996–2001 and in 2003, 2005, 2007, 2010, and 2013. The study was approved by the Human Subjects Committee at Brigham and Women's Hospital and analyses were approved by the Institutional Review Boards at Brigham and Women's Hospital and Boston Children’s Hospital. All methods used in the study followed the best practices for epidemiologic research using observational data and performed in accordance with the relevant guidelines and regulations. [15].

**Fig. 1:**
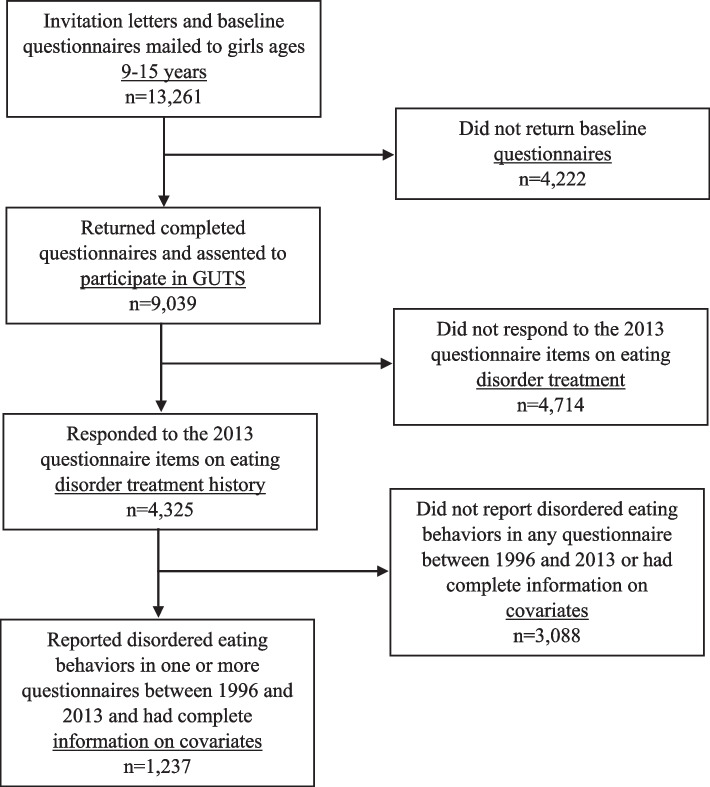
Flow diagram of participants included in the final analytic sample

Figure 1 illustrates a flow diagram of participants included in the final analytic sample. The sample for analysis was restricted to females, based on sex assigned at birth, because there were too few males who received treatment for an eating disorder to conduct a sex-stratified analysis. Women who responded to the 2013 questions on history of treatment for an eating disorder were eligible for inclusion in the analysis (*N* = 4,325). The final analytic sample was further restricted to women who had a low weight and high weight concerns or reported binge eating and/or purging at least monthly in one or more questionnaire between 1996 and 2013 and had complete information on covariates (*N* = 1,237).

### Predictors and correlates

Bulimic behaviors were assessed on all questionnaires. Binge eating was assessed with two questions. The first asked about the frequency of overeating episodes (“During the past year, how often have you eaten so much food in a short period of time that you would be embarrassed if others saw you?”) and then a follow-up question assessing if the participant experienced a loss of control ([During those times], “Did you feel out of control like you couldn't stop eating even if you wanted to stop?”). [16] Purging was assessed by asking how often in the past year the girl made herself throw up or used laxatives to lose weight or to keep from gaining weight. Both the binge eating and purging questions have been validated in the GUTS cohort. [16].

Weight concerns were assess using items from the McKnight Risk Factor Survey. [17] However, these items were not included in the 2010 or 2013 questionnaire. Response options were on a 5-point Likert scale ranging from 1 (“never/not at all”) to 5 (“always/totally”). Girls with a mean score of 4 or greater (i.e., average score of at least “a lot”) were considered to have high weight concerns. [18].

We used the DSM-5 criteria to classify participants at each time-point (i.e., 1998, 1999, etc.) into mutually-exclusive eating disorder profile categories. We classified girls as having symptoms consistent with bulimia nervosa (BN), binge-eating disorder (BED), purging disorder (PD), other specified feeding or eating disorder (OSFED), or anorexia nervosa (AN). Girls who reported binge eating and using vomiting or laxatives for weight control at least weekly were classified as having BN. Those who reported binge eating at least weekly, but no use of vomiting or laxatives for weight control, were classified as having BED. Those who used vomiting or laxatives for weight control at least weekly but did not engage in binge eating were classified as having PD. Girls who engaged in binge eating and/or purging monthly, but not weekly, were classified as having OSFED. Girls who did not engage in binge eating or purging during the age interval were the reference group. Girls who were underweight (as defined below) and reported high levels of weight concerns were classified as possible cases of anorexia nervosa (AN). Since weight concerns were not assessed in 2010 or 2013, the AN proxy could only be constructed for 1996–2007. Although the categories were mutually exclusive in each year, women could have multiple classifications over the course of the study.

Weight and height were self-reported on all questionnaires. Body mass index (weight/height^2^) was calculated from self-reported weight and height. The validity of self-reported weight and height have been investigated in adolescent and young adult populations [19–21] and the results support the use of self-report in epidemiologic studies of associations with weight and BMI, although there is some underestimation with self-report. BMI was used to classify the participants’ weight status into three categories that align with those a clinician might use when assessing body size: “underweight/healthy weight”, “overweight”, or “obese”. For participants < 18 years, we use the international obesity task force pediatric standards developed by Cole and colleagues, [22] which are based on data from six countries. These were developed to map to the adult standards at age 18. These age- and gender-specific BMI cut-off values converge at a BMI of 25 kg/m^2^ or 30 kg/m^2^ at age 18. For participants ≥ 18 years, we use the U.S. Dietary Guidelines [23] classify BMI as follows: < 18.5 kg/m^2^ is “underweight”, 18.5–24.9 kg/m^2^ is the “healthy weight” range, 25–29.9 kg/m^2^ is “overweight”, and ≥ 30 kg/m^2^ is “obese”.

Questions on a diagnosis for anxiety disorder and depression were assessed in 2013. Participants were asked if they had ever been diagnosed by a medical professional with a range of conditions, including depression and anxiety. For each of these disorders the participant was asked to report the time of diagnosis: < 2006, 2006–2008, 2009–2011, or > 2012.

Information on family income was reported by the GUTS participant’s mother in 2001 as part of NHS II. Racial and ethnic group identification was collected in GUTS in 1996.

### Outcomes

On the 2013 questionnaire, participants were asked whether they were currently receiving treatment for an eating disorder or had received treatment for an eating disorder in the past. Participants who reported receiving treatment were also asked the age when they had received treatment: 9–12 years, 13–15 years, 16–18 years, 19–22 years, 23–27 years, or 28 or older. Participants could indicate treatment at multiple ages. The analysis was restricted to treatment that occurred at ages 16 and older because year of diagnoses of depression and anxiety (one of the exposures of interest) was not available at ages younger than 16.

### Statistical method

Logistic regression models were conducted to estimate the relative odds of receiving treatment. We used generalized estimating equations to account for non-independence due to sibling clusters. All analyses controlled for age. Correlates and predictors came from the same age or an earlier time point. In the final models, the variables that were significant in the age-adjusted analyses were included. In addition to focusing the analysis to treatment occurring at ages 16 and older, as described above, the analysis was also restricted to women who engaged in at least monthly binge eating or purging or had high weight concerns despite being underweight in at least one year because this is the group who would be most likely to have a diagnosis and therefore the group most likely to receive treatment. The reference group was participants who were asymptomatic in the specific time period (i.e., not classified as AN, BN, BED, PD, or OSFED). Moreover, we examined the final model both using data from just 1996–2007 as well as from the full 1996–2013 data, since AN symptoms were assessed only up through the 2007 questionnaire.

## Results

In 2013, when history of treatment for an eating disorder was assessed, the mean age of the sample of 1,237 women who reported disordered eating behaviors in at least one questionnaire was 28.0 years, range 25–31 years. The sample was 9- 31 years when the information on eating disorder symptom profiles was assessed, was 93.8% White, and 91.8% had some form of health insurance (Table 1).

**Table 1:** Distribution of age, body mass index (BMI), race/ethnic background and family income among 1237 women in the Growing Up Today Study who engaged in ≥ monthly binge eating or purging or had low weight and high weight concerns in ≥ 1 year

	Mean (S.D.) or percent
Age (years) in 2013	28.0 (1.7)
BMI (kg/m^2^) in 2013	25.7 (6.3)
Racial/ethnic groups	
Hispanic	1.5%
African American/Black	0.7%
Asian	1.5%
White	93.8%
Other	2.5%
Family income	
< $50,000	12.3%
$50,000-$99,999	45.0%
≥ $100,000	42.7%
Eating disorder symptom profile^a^	
Anorexia nervosa	2.8%
Bulimia nervosa	7.8%
Binge-eating disorder	24.8%
Purging disorder	22.6%
OSFED	81.8%
Diagnosis of depression or an anxiety disorder	26.6%

^a^In one or more years between 1996–2007(for anorexia nervosa) or 2010 (all other disorders). Participants could transition between diagnoses over time, so the total is greater than 100%

Among women who reported disordered eating behaviors in at least one questionnaire, approximately 2.8% (*n* = 35) of the women reported the symptoms of AN in at least one year between 1996 and 2007, the final time possible AN cases were able to be detected. Between 1996 and 2013, 96 women (7.8% of those who reported disordered eating in at least one questionnaire) reported the behavioral symptoms of bulimia nervosa (≥ weekly binge eating and purging), 307 (24.8%) reported the behavioral symptoms of BED (≥ weekly binge eating and no purging), 279 (22.6%) reported the behavioral symptoms of PD (≥ weekly purging and no binge eating), and 1,012 (81.8%) reported OSFED in one or more years. Approximately 10.7% of the sample reported receiving treatment for an eating disorder. The percentage who received treatment ranged by symptom profile: 20% of women with symptoms of AN, 43% of women with behaviors of BN, 11% of women with behaviors of BED, 21% of women with behaviors of PD, and 11% of women with OSFED.

In age-adjusted analyses (Table 2), race (odds ratio (OR) = 0.86, 95% confidence interval (CI) 0.34–2.04), family income (OR = 1.12, 95% CI 0.78–1.60), and insurance status (OR = 1.50, 95% CI 0.58–3.86) were not associated with whether a woman received treatment for an eating disorder (and these variables were thus not included in the final model). However, weight status was strongly related to receiving treatment. Women with obesity were significantly less likely than their leaner peers to receive treatment (OR = 0.22, 95% CI 0.09–0.55). The association became stronger when the models were further adjusted for history of a diagnosis of depression or anxiety and eating disorder symptom profiles (OR = 0.19, 95% CI 0.07–0.50).

**Table 2:** Correlates of receiving treatment for an eating disorder among women in the Growing Up Today Study who were symptomatic for an eating disorder in at least one year

	Age-adjusted	Final model using eating disorder data 1996–2007 (the last AN assessment)	Final model using eating disorder data 1996–2013
Race (White vs. non-White)	0.86 (0.36–2.04)		
Family income	1.12 (0.78–1.60)		
Insurance	1.50 (0.58–3.86)		
Weight status			
Healthy or underweight	Ref	Ref	Ref
Overweight	0.82 (0.53–1.27)	0.84 (0.51–1.38)	0.84 (0.52–1.35)
Obese	0.22 (0.09–0.55)	0.13 (0.04–0.45)	0.19 (0.07–0.50)
Diagnosis of anxiety or depression	3.19 (2.16–4.71)	2.90 (1.81–4.63)	2.80 (1.86–4.21)
Symptom profile			
Anorexia nervosa	5.29 (1.91–14.64)	5.13 (1.84–14.29)	
Bulimia nervosa	12.01 (7.05–20.47)	12.38 (7.30–21.00)	13.03 (7.95–21.35)
Binge eating disorder	1.96 (1.07–3.60)	2.09 (1.14–3.82)	1.79 (0.99–3.23)
Purging Disorder	3.44 (2.00–5.92)	3.40 (2.00–5.79)	3.15 (1.92–5.15)
OSFED	0.79 (0.35–1.81)	0.85 (0.37–1.94)	0.91 (0.42–1.95)

**Fig. 2:**
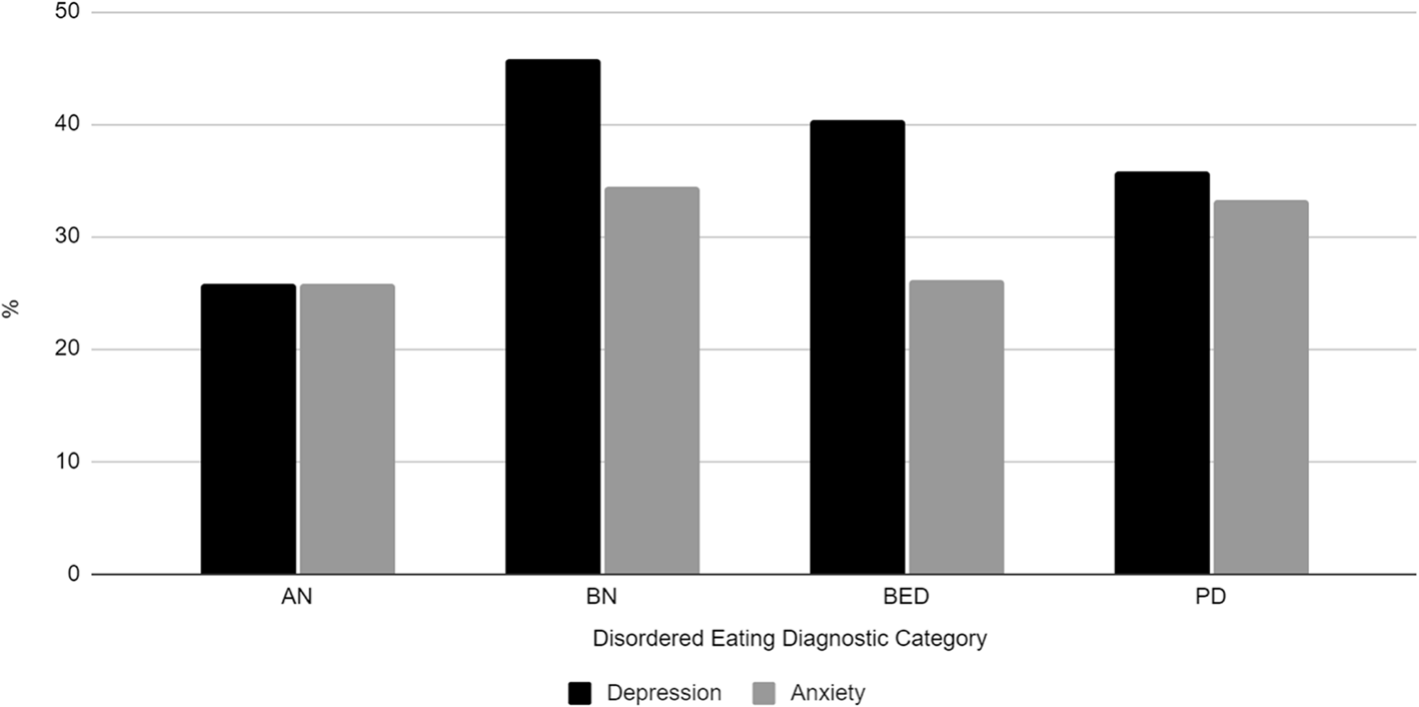
Comorbidity of self-reported depression and anxiety diagnoses by eating disorder symptom profile among women in the Growing Up Today Study

Approximately 32.9% of the sample reported a diagnosis of depression and 25.7% reported a diagnosis of anxiety. Among those who received a diagnosis of depression, 58.2% reported also receiving a diagnosis of an anxiety disorder. A history of a diagnosis of depression was most prevalent among women who had a symptom profile of BN or BED (Fig. 2). Regardless of symptom profile, women with a diagnosis of depression or an anxiety disorder were significantly more likely to receive treatment for an eating disorder (OR = 3.19, 95% CI 2.16–4.71) than their peers without a diagnosis of depression of anxiety (Table 2). As expected, eating disorder symptom profile was strongly associated with receiving treatment for an eating disorder. Women with the BN symptom profiles were the most likely to receive treatment (OR = 12.01, 95% CI 7.05–20.47). Whereas, women with the symptom profile of BED, the most prevalent full criteria disorder, or OSFED were the least likely group with an eating disorder to receive treatment.

## Discussion

Among a population-based sample of predominantly middle-class White women living throughout the United States, rates of receiving treatment for an eating disorder were low. Despite more than 90% of the women reporting that they had health insurance, less than half of women who engaged in frequent binge eating and/or purging received treatment for an eating disorder. The rates of receiving treatment were lowest in the most common eating disorders, binge-eating disorder and purging disorder.

Unlike previous studies, we did not observe differences by socio-economic status, [8] which may reflect that our sample was predominantly middle-class women. Similar to Sonneville and Lipson, [8] we did not observe that race or ethnic group was related to receiving treatment. However, our sample included very few non-White participants, and future research should continue to examine this question among more racially and ethnically diverse samples. In contrast, Falvey et al., [24] using a large cross-sectional sample of college and graduate students in the United States and Canada, observed that Asian and Hispanic students were more likely than White students to have symptoms of an eating disorder, but less likely to receive a diagnosis.

There are a range of factors that can influence whether a person receives treatment, including perceived need, stigma of mental health, negative attitudes about treatment for a mental health disorder, lack of knowledge about treatment, and cost. [25, 26] These attitudinal and financial barriers have an enormous impact on determining who receives treatment. For example, among 2,822 college students in one large study, only 53% of the women who scored high on the SCOFF eating disorder screening instrument perceived that they had a need for treatment and only 22% had received treatment. [27] Although there is considerable heterogeneity across studies, overall, it appears that stigma about mental health is higher among Asian, Black, and Hispanic adults compared to White adults. [7] The results suggest that new approaches are needed to change perceptions about mental health disorders and their treatment, particularly among ethnic and racial minority populations.

A potentially easier barrier to address is clinician misconceptions. Our results and those of Sonneville and Lipson [8] demonstrates that women with lower body weights were more likely to be diagnosed [8] or receive treatment compared to women with higher body weights. These findings suggest that clinicians may screen for an eating disorder among underweight patients, but not among higher weighted patients. Although the immediate health consequences of AN may be more severe than that of other eating disorders, AN is less common than BN or BED, [28, 29] and BN and BED are associated with substantial impairment. [12] Moreover, obesity is substantially more common than underweight [30] and people with obesity are the most likely to have an eating disorder. [5].

There are several important implications from our findings. First, clinicians must be educated about the symptoms, prevalence, and need to screen for PD, BED, and OSFED, particularly among patients with obesity. Second, more needs to be done to decrease barriers to eating disorder treatment. Third, results from treatment-seeking samples of women with eating disorders should not be generalized to eating disorders in the general population. Generalizing findings related to BED or OSFED from treatment-seeking samples could be highly misleading since less than 15% of the probable cases received treatment. There is accumulating research which suggests that treatment-seeking samples are a biased representation [31] and may overestimate rates of psychiatric comorbidity and underestimate the burden of eating disorders among people with obesity. These biases have important implications for drawing inference about associations with mental and physical health conditions. In order to better understand sex, race, ethnic group, and weight status associations, it is essential to use population-based samples.

There are several limitations to our study to keep in mind when drawing inference. These include the small number of low SES or non-White women, the use of a self-report instrument to assess eating disorders, and our inability to assess AN after 2007 because weight concern questions were not assessed. Our findings may not be generalizable to non-White individuals, males, or more socio-economically diverse populations. An additional limitation is that our outcome was eating disorder treatment occurring at or after age 16 years. We chose this analytical approach since there was no information on diagnoses of anxiety or depression before age 16, and as such, we would have run the risk of reverse causation if we examined associations of anxiety/depression diagnoses with eating disorder treatment occurring before age 16. However, our approach may not fully capture the full effect of the correlates on eating disorder treatment occurring at all ages and our findings should be interpreted specifically to treatment occurring after age 16 and into young adulthood. We additionally had a relatively small sample of women with symptom profiles of AN (*n* = 35) and BN (*n* = 96), therefore, the confidence intervals for those associations were wide. It is possible that patterns have changed since 2013, however, obesity bias remains widespread thus it is likely that the under-detection in people with obesity persists. Moreover, we were unable to identify women who met criteria for atypical AN, such as those who engaged in AN behaviors but were not underweight, and future research should examine predictors and correlates of treatment among these individuals as well, especially weight. Lastly, our complete case analysis approach may result in selection bias if being excluded due to missing data was related to the outcome and exposures of interest. The strengths of the study include the use of a large sample living throughout the United States, the numerous waves of data collection (1996–2013), and the population-based sample offset some of the limitations.

Future work will need to directly assess how clinicians make decisions regarding screening for eating disorders and educating them about BED, PD and other types of OSFED. The majority of adolescent and emerging adult women who are symptomatic for an eating disorder do not receive treatment. Those with obesity are less likely to receive treatment. Clinicians should assess women across the weight range for eating disorder symptoms to increase identification of cases and lessen the weight-status disparity.

## Data Availability

The data that support the findings of this study are available from The Channing Division of Network Medicine, but restrictions apply to the availability of these data, which were used under license for the current study, and so are not publicly available. Data are however available from the authors upon reasonable request and with permission of the Channing Division of Network Medicine. Outside investigators can receive access to data from the GUTS in one of three ways: 1) through the GUTS Data Repository, the external investigator is granted a login to our computer system, accesses and analyzes our data, 2) the external investigator collaborates directly with a GUTS investigator and programmer who conduct the analyses on the external investigator’s behalf, or 3) a specific, limited dataset is created to send to the external collaborator. Given the greater flexibility provided by the first option with direct access to all cohort data, the second and third options are not utilized as frequently, except in the case of consortia projects pooling data from multiple cohorts.
